# Mu rhythm suppression reflects mother-child face-to-face interactions: a pilot study with simultaneous MEG recording

**DOI:** 10.1038/srep34977

**Published:** 2016-10-10

**Authors:** Chiaki Hasegawa, Takashi Ikeda, Yuko Yoshimura, Hirotoshi Hiraishi, Tetsuya Takahashi, Naoki Furutani, Norio Hayashi, Yoshio Minabe, Masayuki Hirata, Minoru Asada, Mitsuru Kikuchi

**Affiliations:** 1 Research Center for Child Mental Development, Kanazawa University, Kanazawa 920-8640, Japan; 2 Department of Adaptive Machine Systems, Graduate School of Engineering, Osaka University, Suita, 565-0871, Japan; 3 Department of Neurosurgery, Osaka University Medical School, Suita, 565-0871, Japan; 4 Department of Psychiatry and Neurobiology, Graduate School of Medical Science, Kanazawa University, Kanazawa 920-8641, Japan; 5 School of Radiological Technology, Gunma Prefectural College of Health Sciences, Maebashi, 371-0052, Japan

## Abstract

Spontaneous face-to-face interactions between mothers and their children play crucial roles in the development of social minds; however, these inter-brain dynamics are still unclear. In this pilot study, we measured MEG mu suppression during face-to-face spontaneous non-linguistic interactions between mothers and their children with autism spectrum disorder (ASD) using the MEG hyperscanning system (i.e., simultaneous recording). The results demonstrated significant correlations between the index of mu suppression (IMS) in the right precentral area and the traits (or severity) of ASD in 13 mothers and 8 children (MEG data from 5 of the children could not be obtained due to motion noise). In addition, higher IMS values (i.e., strong mu suppression) in mothers were associated with higher IMS values in their children. To evaluate the behavioral contingency between mothers and their children, we calculated cross correlations between the magnitude of the mother and child head-motion during MEG recordings. As a result, in mothers whose head motions tended to follow her child’s head motion, the magnitudes of mu suppression in the mother’s precentral area were large. Further studies with larger sample sizes, including typically developing children, are necessary to generalize this result to typical interactions between mothers and their children.

The social interaction between a mother and her child has a crucial role in childhood development and is critical in the development of social minds. The cognitive and emotional interactions between a mother and her child are induced by their behaviors, and such brain-to-brain interactions must play a crucial role in forming social minds. However, the neural correlates underlying this process are unclear. Only a few papers have reported on this question, and they have all either assessed only the mother’s responses or only the child’s responses during mother-child interactions^1^,^2^.

To better understand the inter-brain dynamics that occur during human interactions, it is necessary to research not only a single brain in isolation but two brains during a real interaction. Simultaneous multiple brain functional measurements may reveal the brain dynamics underlying social interactions. For the study of multiple adult brains during an interaction, hyperscanning methods have already been applied in previous electroencephalography (EEG), functional magnetic resonance imaging (fMRI), or near infrared spectroscopy (NIRS) studies^3^,^4^,^5^,^6^,^7^,^8^,^9^. However, to date, brain-to-brain interactions between mothers and their children have not yet been reported. Magnetoencephalography (MEG) is a child-friendly device that provides high-resolution spatiotemporal dynamics of neuromagnetic fields during various cognitive and behavioral activities^10^,^11^. Therefore, hyperscanning MEG has considerable potential for the study of brain-to-brain interactions between mothers and their children^12^.

It has been suggested that mu suppression over sensory-motor regions reflects a resonance system in the human brain analogous to mirror neurons in monkeys^13^. Recently, several studies of typically developed participants linked EEG mu suppression to higher social information processing, including social skills^14^, theory of mind^15^ and empathy^16^,^17^. Therefore, mu suppression was predicted to reflect not only the individual’s motor representations but also the social mind^18^. Intriguingly, two recent studies demonstrated aberrant responses of mu rhythm in adults^19^ and children^20^ with autism spectrum disorder (ASD), which appears in early childhood, causing delays or impairments in social interactions and communication^21^. The term “spectrum” is crucial to understanding autism because of the wide range of symptom severity. Given that the soundness of social minds are reflected in the degree of mu suppression, the dynamics of mu rhythms during real communication between mothers and their children might serve as an index of soundness in brain-to-brain interactions.

In this study, typically developed mothers and their young children with ASD (with various levels of social ability) were included to test a high diversity of mother-child interactions. Although these mother-child interactions are not typical, we believed a larger diversity of participants would better reveal the neural correlates of social behavior.

The first aim of this pilot study was to investigate whether mu suppression induced by face-to-face non-linguistic spontaneous interaction (Fig. 1) reflects sociality in typically developed mothers and their children with ASD. The second aim of this study was to investigate whether the degree of such mu suppression is similar between the pair (i.e., does a larger mu suppression in the mother correlate with a larger mu suppression in her child?). The third aim of this study was to investigate whether the degree of mu suppression is correlated with the temporal behavioral contingency between a mother and her child during face-to-face non-linguistic spontaneous interactions.

## Results

MEG recordings were completed for all 14 pairs. However, we excluded the MEG data of 5 children due to excessive motion noises, and excluded the MEG data of one mother due to too much magnetic noise due to the presence of dental metals. In these excluded subjects, we could not obtain a sufficient period of noise-free MEG data (i.e., we captured less than a 50 sec period). Eventually, as shown in Table 1, we analyzed the MEG data from 13 mothers and 8 children (4 females and 4 males) whose number of noise-free MEG epoch (2.5 sec) were more than 20 (i.e., more than 50 sec) for both the “Live” and “DVD” conditions. For the mother-child correlation study and for the analysis of the mother-child behavioral interactions, only the 8 children and their mothers were included. The participating children were between the ages of 48 and 94 months, and their mothers were between 31 and 41 years of age (Table 1). All of participants were right-handed.

### Correlation between the index of mu suppression and the presence of autistic traits

From the results of Spearman’s rank correlation coefficients analysis of mothers’ data, significant negative correlation was found between the IMS in the right precentral area and AQ total score (n = 13, *ρ* = −0.78, *p* = 0.002) (Fig. 2a). Excluding one outlier still resulted in significance (n = 12, *ρ* = −0.73, *p* = 0.007). After controlling for confounding variables (i.e., suppression of the same frequency range in the visual cortex), this significance was still maintained (n = 13, *ρ* = −0.64, *p* = 0.032). In the left precentral area, there were no significant correlations between the IMS and the AQ scores (*ρ* = −0.16, *p* > 0.05).

Spearman’s rank correlation analysis of the children’s data revealed a significant negative correlation between the IMS of 10–12 Hz in the right precentral area and the SRS total *T*-score (*ρ* = −0.76, *p* = 0.028) (Fig. 2b), whereas no significance was found for the IMS of 8–10 Hz (*ρ* = −0.45, *p* > 0.05). After controlling for confounding variables (i.e., suppression of the same frequency range in the visual cortex and age in month), this significance was still maintained for the IMS of 10–12 Hz (*ρ* = −0.88, *p* = 0.047). In the left precentral area, no significant correlations were observed for the IMS of 8–10 Hz (*ρ* = −0.55, *p* > 0.05) and 10–12 Hz (*ρ* = −0.41, *p* > 0.05).

In the present study, as an additional exploratory analysis, we added the correlation analysis between the IMSs in all brain areas corresponding to the 68 freesurfer Desikan-Killiany atlas regions and the AQ total scores in the mothers (*n* = 13) or the SRS total scores in the children (*n* = 8). As shown in Fig. 3, the whole brain *ρ* value map showed that autistic traits were negatively correlated with IMS, mainly in the right precentral area both in mothers and children. That is, fewer ASD traits in mothers and children were associated with robust mu suppression in the right precentral area during their face-to-face interactions.

### Correlation of mu suppression between mothers and children

As shown in Figure S1, there was a significant correlation between the mothers and children in terms of the IMSs in the right precentral area (*ρ* = 0.71, *p* = 0.047). By contrast, there was no significant correlation between the IMSs of mothers and children in the left precentral area (*ρ* = 0.31, *p* > 0.05).

### Mu suppression and behavioral contingency between mothers and children

As shown in Fig. 4, we obtained 8 time series of cross correlation coefficients for the mother and child head motion comparisons from 8 mother-child pairs.

In the mothers (Table S1), Spearman’s rank correlation analysis revealed a significant positive correlation between IMS in the right (ρ = 0.80, *p* = 0.017) or left (*ρ* = 0.73, *p* = 0.040) precentral area and a latency of the peak in the cross correlation. Therefore, in mothers whose head motions tended to follow their child’s head motions during face-to-face interaction, the magnitude of mu suppression in the mother’s precentral area was large (Figure S2). In addition, Spearman’s rank correlation analysis revealed a significant positive correlation between IMS in the left (*ρ* = 0.74, *p* = 0.037) but not the right (*ρ* = 0.19, *p* > 0.05) precentral area and a peak value of cross correlation coefficient. Therefore, in a mother whose head motion was highly associated with her child’s head motion during face-to-face interactions, the magnitude of mu suppression in the mother’s left precentral area was large.

In children (Table S1), Spearman’s rank correlation analysis failed to reveal any significant correlation between the IMS and any indices of cross correlation coefficients (*p* > 0.05).

## Discussion

This is the first study to demonstrate that the degree of mu suppression during face-to-face spontaneous, non-linguistic mother-child interactions is negatively correlated with the degree of autistic traits in the mother and with symptom severity in children with ASD. In previous studies, mu suppression has been used as an index of perception–action coupling involving the mirror neuron system (MNS)^22^,^23^. In addition, recent studies have demonstrated that the mu rhythm changes in response to the observation of not only biological motions but also emotional facial expressions^24^ and oro-facial movements^25^. In previous studies, diminished mu modulation during action observation in individuals with ASD has been interpreted as a dysfunction of simulation networks such as the MNS^19^,^20^,^26^,^27^. Although some EEG studies did not find such mu dysfunction in the ASD subjects^28^,^29^, the segregation of the mu band into two sub-bands revealed an abnormal response in the upper mu sub-band of the ASD subjects^19^,^20^. Our results from upper mu sub-band during the face-to-face interactions were in-line with the findings of previous studies. In addition, the present study represents the first study to demonstrate that a diminished response of the mu sub-band to the face-to-face spontaneous interactions were associated with higher traits of ASD in mothers or with more severe ASD symptoms in their children.

A unique feature of this study is that we focused on the brain activities during task free, face-to-face, non-linguistic, and spontaneous mother-child interactions. It is impossible to reproduce the same experimental conditions (i.e., spontaneous interaction) for each pair; therefore, the hyperscanning MEG system was essential in the present study^12^. Through this hyperscanning system, we demonstrated a significant correlation between the mothers and their children in their IMS in the right precentral area. Because of our experimental design, we could not draw any definitive conclusions regarding the underlying mechanisms of this result. However, there are three possible interpretations of this result. First, it is possible that the similarity of the brain traits concerning social information processing between the mothers and their children contributed to our results (i.e., this could be a trait-dependent phenomenon). A second interpretation is that, regardless of the similarity of brain traits between the mothers and children, the quantity and/or quality of the face-to-face mother-child interaction during the MEG measurement contributes to the results by chance (i.e., as a state-dependent phenomenon). Another possible interpretation is that both these mechanisms are at play (i.e., both traits and states could contribute to these results).

With regard to the behavioral contingency (or resonance) between a mother and her child during their face-to-face interactions, 5 of the 8 mother’s head motions tended to follow their child’s head motions and only one mother’s head motions tended to lead her child’s head motions (Fig. 4). Intriguingly, it is well known that human beings tend to unconsciously imitate the behavior of others and that the individual who is imitated tends to have good impression on the imitator. This is called the chameleon effect^30^. Furthermore, the effects of the behavioral imitations are not simply due an increased preference for the imitator, but are instead due to an increased prosocial orientation^31^. Our results suggested that in mother-child interactions, the mothers unconsciously tend to act to let their children increase their prosocial orientation. In addition, in mothers whose head motions tended to follow their children’s head motions with a larger time lag, the magnitudes of mu suppression in their precentral areas were larger. This result seems to be natural. Mothers who take a behavioral sequence of perception-actions can observe their children’s motion well, and therefore, their own motor resonance systems may be more activated. This is the first study to demonstrate that the quality and quantity of mother-child interactions (i.e., for typically developed mothers and children with ASD) under the experimental conditions are reflected in the mu suppression.

This study has some limitations. First, we calculated the index of mu suppression as follows: the magnitude of mu oscillations during the “Live” condition was subtracted from those during the “DVD” condition. During the “DVD” condition, it is possible that differences in the measured mu activities may be related to the different spatiotemporal properties of the DVD stimuli during the periods corresponding to the selected artifact-free segments. Second, we did not quantify how psychologically engaged or disengaged the children were with their DVDs. This uncertain baseline condition (i.e., DVD condition) may affect IMS in the present study. Third, a recent EEG study reported “the mu suppression can be used to index the human MNS, but the effect is weak and unreliable and easily confounded with alpha suppression in occipital area”^32^. Therefore, mu suppression as an index of the human MNS remains open to debate. Fourth, the ASD children with various SRS scores participated in the present study; however, we did not include typically developing children. Therefore, the findings may not apply to mothers and typically developing children. Fifth, we recorded the head positions of the subjects using video monitors during the MEG recordings, and we eliminated MEG data from power analysis by visual inspection if the head position of the subject had obviously moved from its starting position. Further study using a quantification algorithm for head movement will provide more reliable data.

In conclusion, to the best of our knowledge, this is the first study to simultaneously study the neuromagnetic activities in both mothers and their children with ASD during task-free face-to-face spontaneous interactions. Our results from pilot study demonstrated that the mu suppression level of ASD children and their mothers reflects their social ability or autistic traits and demonstrated a correlation between the mu suppression in the mothers and their children. Further studies with larger sample sizes, including both typically developing children and children with ASD, are necessary to test the reliability of these findings and support their generalization to interactions between typically developed mothers and typically developing children.

## Method

### Participants

Fourteen children with ASD and their mothers participated in this experiment. All child participants were recruited from Kanazawa University’s Hospital and prefectural hospitals in Toyama. They were diagnosed by a clinical psychiatrist and a clinical psychologist with more than 5 years of experience in ASD using the Autism Diagnostic Observational Schedule–Generic (ADOS)^33^, the Diagnostic Interview for Social and Communication Disorders (DISCO)^34^, and the DSM-5^35^ criteria at the time that they entered into this study. All children were included in this study when they fulfilled the diagnosis of childhood autism, atypical autism or Asperger’s syndrome with DISCO, or met the ADOS criteria for the autism spectrum. Exclusion criteria for children included known hearing loss or a central nervous system involvement other than autism.

Mothers had no prior or current developmental, learning, or behavioral problems. If the mothers reported difficulties in their daily life because of their intelligence level, we excluded them from this experiment.

The mothers agreed to their child’s and their own participation in the study with full knowledge of the experimental nature of the research. Written informed consent was obtained prior to participation. The Ethics Committee of the Kanazawa University Hospital approved all methods, and all procedures were performed in accordance with the Declaration of Helsinki.

### Psychological tasks for autistic traits

Quantitative autistic traits in the enrolled children were assessed by the parents using the Japanese version of the Social Responsiveness Scale (SRS)^36^,^37^. Higher scores on the SRS indicate a higher degree of social impairment. The raw scores of the SRS were converted to *T*-scores (with a mean of 50 and a standard deviation of 10). A *T*-score of 60 through 75 is interpreted as falling in the mild to moderate range and considered typical for children with mild or “high functioning” ASD, whereas a *T*-score of 59 or less suggests an absence of ASD symptoms. The Kaufman Assessment Battery for Children (K-ABC)^38^ was employed to estimate the intelligence levels of the children. Traits of ASD in the mothers were assessed by the Autism Spectrum Quotient (AQ)^39^, which consisted of self-reported measures of autistic traits. This questionnaire can be used to assess milder variants of autistic-like traits (i.e., a high AQ) in typically developing individuals^40^,^41^.

### Magnetoencephalography recordings

#### Hyperscanning MEG system

Two MEG systems were housed in a magnetically shielded room (Daido Steel, Nagoya, Japan) at the Yokogawa Electric Corporation. One was a 160 channel Superconducting Quantum Interference Device (SQUID) consisting of a whole-head gradiometer equipped with coaxial type gradiometers (MEG vision NEO, Yokogawa Electric Corporation, Kanazawa, Japan). This MEG system was used to record the mother’s neuromagnetic activities. The other MEG system was a 151 channel whole-head gradiometer equipped with coaxial type gradiometers (PQ1151R, Yokogawa Electric Corporation/KIT, Kanazawa, Japan), which was designed to measure a child’s or infant’s neuromagnetic activities^42^,^43^,^44^,^45^,^46^,^47^. The custom child-sized MEG system allowed for measurements of the brain responses in children and infants, which would be difficult to obtain with conventional adult-sized MEG systems. To measure the mother-child interactions in a face-to-face situation, we set up a real-time dual video presentation to show the facial expressions of the mother and child, in addition to a standard auditory recording and stimulation system^12^. This video presentation system allows the mother and her child to see each other’s facial expressions in real time. Details of our system and experimental method are presented in a previous study^12^. Data acquisition detail was shown in Supplementary material.

#### Experimental procedures

In this study, the mother and child lay supine on a bed, each facing a half-mirror screen. One staff member (author Y.Y.) remained in the shielded room to confirm that each participant was concentrating on the task and to encourage the children to maintain a steady body position when necessary. Short movies and the other’s facial expression with a live movie or a still picture were alternately displayed for 10 or 15 sec, respectively, on the half-mirror screen, while simultaneous, neuromagnetic recordings of their brain activities were performed. In the facial expression periods (i.e., the live face-to-face condition), the mother and her child looked at each other. We instructed them not to speak and not to move their head or body (e.g., “Look at each other’s face. Do not move your head and body, as much as possible.”). Their facial expressions were not restricted. We did not encourage or restrict interaction with each other (i.e., we tried not to interfere with voluntary communication). In the short movie periods, the mother and her child looked at the same short movies that were selected according to the child’s interest. The participants were carefully monitored using a video monitoring system to assess their compliance with the instructions and record any notable artifacts, such as head motion, inappropriate head position, and consistently attending to the mother’s projections.

#### Index of mu suppression

**Figure 1:**
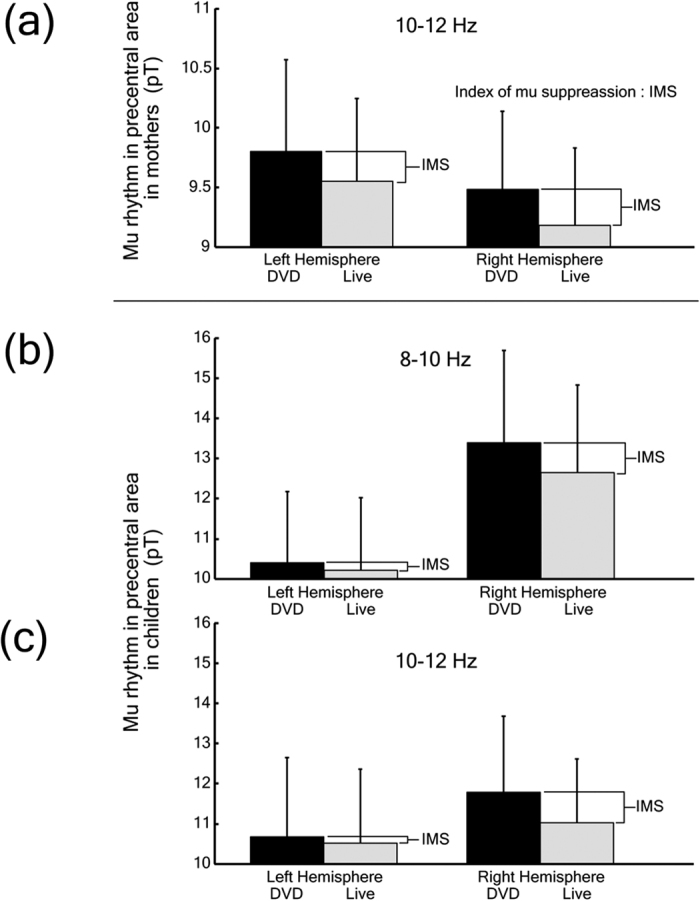
Mu rhythm amplitude in precentral areas of mothers and children. (**a**) mu rhythm (10–12 Hz) amplitude in the precentral area in mothers (*n* = 13). (**b**) mu rhythm (8–10 Hz) amplitude in the precentral area in children (*n* = 8). (**c**) mu rhythm (10–12 Hz) amplitude in the precentral area in children (*n* = 8). The black bar indicates the DVD condition. The gray bar indicates the face-to-face condition. The index of mu suppression (IMS) was calculated using the following formula: IMS = mu rhythm power (DVD - Live). The error bar represents the standard error of the mean.

**Figure 2:**
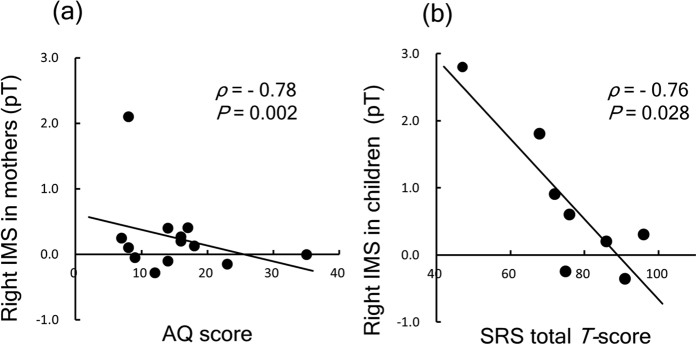
Scatter plot of the IMS in right hemisphere and the autistic traits. (**a**) Scatter plot of the IMS in the right hemisphere and the AQ score in the mothers. Spearman’s rank correlation coefficient was significant (*n* = 13, ρ = 0.78, *p* = 0.002). (**b**) Scatter plot of IMS in the right hemisphere and the SRS total *T*-score in children. Spearman’s rank correlation coefficient was significant (*n* = 8, *ρ* = 0.76, *p* = 0.028).

**Figure 3:**
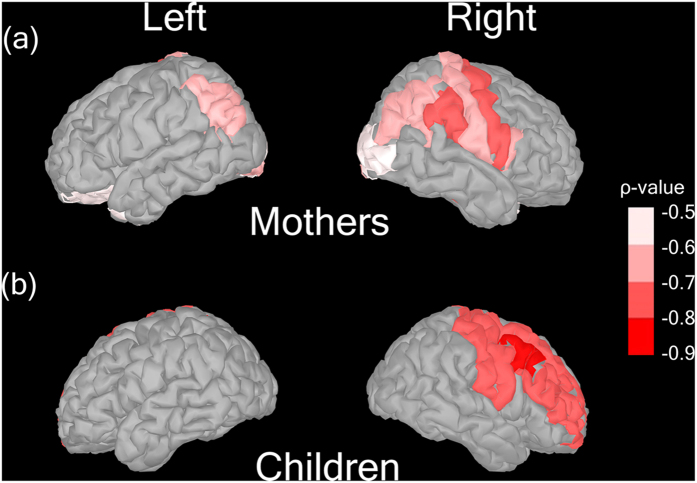
Whole-brain statistical maps of IMS. From the results of Spearman’s rank correlation analysis between the IMSs in all brain areas corresponding to 68 freesurfer Desikan-Killiany atlas regions and the AQ total score (in the mothers) or SRS total *T*-scores (in the children), a significant correlation was found in the brain area located close to the central sulcus in the right hemisphere (*p* < 0.05) in mothers (**a**) and children (**b**). The color bar indicates *ρ*-values.

**Figure 4:**
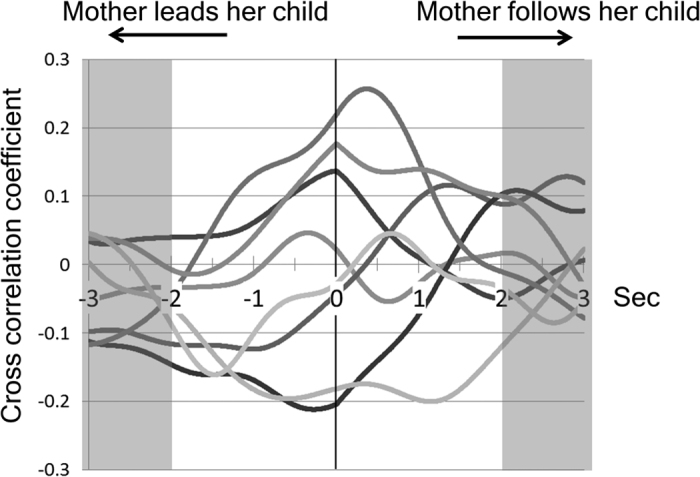
Cross-correlation coefficients in 8 mother-child pairs. Data represent the cross-correlation coefficients of each of the 8 mother-child pairs. The ordinate axis represents the cross-correlation coefficient, and the abscissa represents the directionality. A positive peak value of the correlation coefficient in the abscissa axis indicates that the “mother tends to follow her child”, a negative peak value indicates that “mother tends to lead her child”, and a 0 indicates that there is a “tendency for no time-lag between the head motions of the mothers and their children”.

Mu rhythms have typically been identified in previous EEG studies as 8–13 Hz oscillations^48^ (Fig. 1). In addition, mu rhythms have been thought to functionally segregate into two discrete sub-bands^49^,^50^. Intriguingly, two recent studies demonstrated aberrant responses to the observation of human actions in adults^19^ and children^20^ with autism spectrum disorder (ASD) in the upper sub-band. Based on these previous studies, for the mothers, we focused on suppression in the upper mu band (10–12 Hz) (Fig. 1a) in the precentral area in both hemispheres. For the children, due to uncertainty regarding the frequency range corresponding to the upper mu band reported in adults, we focused on suppression in both the 8–10 (Fig. 1b) and 10–12 Hz (Fig. 1c) bands. We calculated the index of mu suppression as follows: the magnitude of the mu band oscillation in the precentral areas during the “Live” condition (i.e., the face-to-face interactive condition) was subtracted from those during the “DVD” condition. In the present study, higher values indicate a large suppression and lower values indicate a lower suppression. MEG source localization methods were shown in Supplementary material.

#### Analysis of the behavioral contingency between mother and child

For the eight mother-child pairs in which the analysis of mu suppression could be conducted, we analyzed the behavioral contingency between mother and child during face-to-face interactions (i.e., the whole period of the “Live” condition, including motion noise). To quantify the mutual face-to-face interactions between mother and child, we calculated the correlation coefficient sequence between the magnitudes in the mother and child head motions (Supplementary movie S1) (e.g., nod, nod back, nod no, etc.). This procedure was shown in Supplementary material.

### Statistical analysis

We tested our hypothesis that (1) the index of mu suppression (i.e., the magnitude of the mu band oscillation in the right and left precentral areas during the “Live” condition was subtracted from during the “DVD” condition) would correlate with their autistic traits, and (2) the index of mu suppression would correlate between the mothers and children. Furthermore, (3) the contingency of the actual head movements in the mothers and children during the MEG recordings would be correlated with their index of mu suppression.

First, to evaluate the relationship between the index of mu suppression and the presence of autistic traits (AQ in mothers, SRS in children), we performed Spearman’s rank correlation analysis in the mothers (*n* = 13) and children (*n* = 8). If a significant correlation was found, these correlation coefficients were calculated again controlling for confounding variables (e.g., suppression of the same frequency range in the visual cortex). Second, Spearman’s rank correlation was used to investigate the correlation between the mothers and children for the index of mu suppression in eight pairs. Third, we calculated Spearman’s rank correlation coefficients between the index of mu suppression and indices of contingency (i.e., strength and direction of contingency) in eight pairs. All statistical analyses were completed using SPSS Statistics 23 (IBM, NY, USA). We planned this pilot study to show feasibility and to collect data for planning a larger study. Because of the small sample size (i.e., low statistical power), the significance level was set at 0.05, and we did not employ correction for multiple comparisons.

## Additional Information

**How to cite this article**: Hasegawa, C. *et al*. Mu rhythm suppression reflects mother-child face-to-face interactions: a pilot study with simultaneous MEG recording. *Sci. Rep.* **6**, 34977; doi: 10.1038/srep34977 (2016).

## Supplementary Material

Supplementary Information

Supplementary Information

## Figures and Tables

**Table 1:** Demographic characteristics of all participants.

Number of subjects	Mothers (*n* = 13)	Mothers (*n* = 8)	Children (*n* = 8)
Gender (male/female)	—	—	4/4
Chronological age (range)	37.5 years (31–41)	37.8 years (31–41)	74.1months (48–94)
K-ABC mental processing scale (±*SD*)	—	—	95.3 (23.4)
K-ABC achievement scale (±*SD*)	—	—	103.7 (25.0)
Head circumference (range)	—	—	51.2 cm (48.6–53.1)
SRS total score (range)	—	—	76.4 (47–96)

K-ABC, Kaufman Assessment Battery for Children. SRS, Social Responsiveness Scale.
