# Supplementary material for: Mu rhythm suppression reflects mother-child face-to-face interactions: a pilot study with simultaneous MEG recording
Source: Sci Rep. 2016 Oct 10;6:34977. doi: 10.1038/srep34977 (PMC5056356; doi:10.1038/srep34977)
Supplement: Supplementary Information [file srep34977-s1.pdf]

Supplementary material (one legend for movie, two figures, one table and methods)

Scientific Reports: Supplementary materials

Title: Mu rhythm suppression reflects mother-child face-to-face interactions: a pilot study with simultaneous MEG recording

Running Title: mu rhythm in mother-child interactions

Authors: Chiaki Hasegawa,[1] Takashi Ikeda,[3, 5] Yuko Yoshimura,[1] Hirotoshi Hiraishi,[1] Tetsuya Takahashi,[1, 2] Naoki Furutani, [2] Norio Hayashi,[4] Yoshio Minabe,[1, 2] Masayuki Hirata,[5] Minoru Asada,[3] Mitsuru Kikuchi [1, 2*]

1) Research Center for Child Mental Development, Kanazawa University, Kanazawa 920-8640, Japan

2) Department of Psychiatry and Neurobiology, Graduate School of Medical Science, Kanazawa University, Kanazawa 920-8641, Japan

3) Department of Adaptive Machine Systems, Graduate School of Engineering, Osaka University, Suita, 565-0871, Japan

4) School of Radiological Technology, Gunma Prefectural College of Health Sciences, Maebashi, 371-0052, Japan

5) Department of Neurosurgery, Osaka University Medical School, Suita, 565-0871, Japan

Legend for supplementary movie S1.

Movie S1. Head-motion-graphy (HMG).

The magnitudes of the head motions were quantified using motion artifacts in the MEG signals. As shown in this movie, HMG (right movie) practically reflected the head motions (left movie).

Figure S1

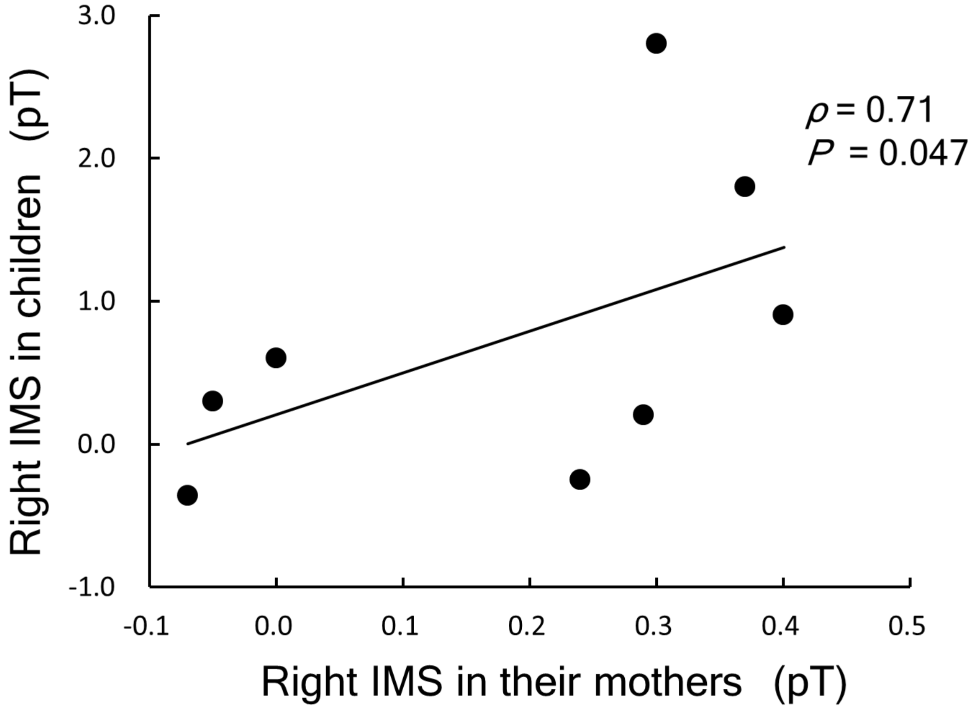

Figure S1. **Scatter plot of IMS in the right hemisphere of mothers and their children.**

Spearman’s rank correlation analysis revealed a significant correlation in the IMS between children and their mothers (n = 8, $\rho = 0.71$, $P = 0.047$).

Figure S2

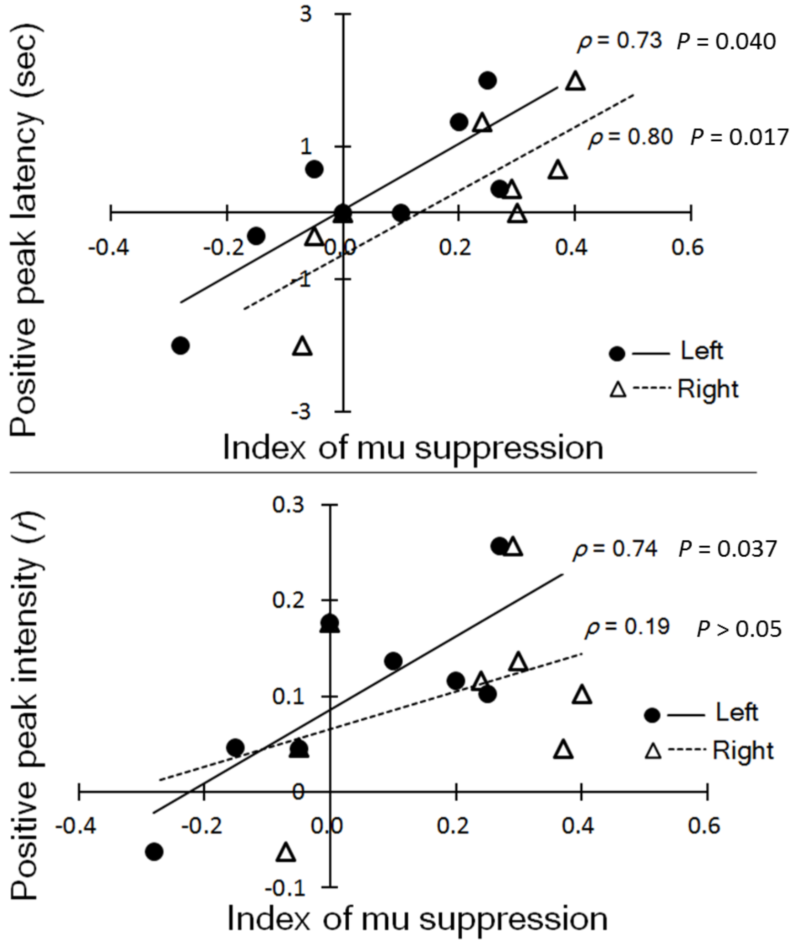

Figure S2. Scatter plot of peak value in the cross-correlation coefficient and IMS in mothers. a, Scatter plot of the positive peak latency of cross correlation coefficients and IMS. Spearman's rank correlation analysis revealed a significant correlation between the left (n = 8, ρ = 0.73, P = 0.040) and right hemispheres (n = 8, ρ = 0.80, P = 0.017). b, Scatter plot of the positive peak values in the cross correlation coefficient and IMS. A significant correlation was found in the left (n = 8, ρ = 0.74, P = 0.037) hemisphere but not in the right hemisphere (n = 8, ρ = 0.19, P > 0.05). The closed circles indicate the value corresponding to the left hemisphere, and the open triangles indicate the value corresponding to the right hemisphere. Solid line: regression line for the left hemisphere; broken line: regression line for the right hemisphere.

Table S1. Spearman's rank correlation coefficients (ρ) between latency/intensity in positive peak (within 2sec) with index of mu suppression in precentral area in mothers and her children (n = 8).

Participant	Hemisphere	Positive peak latency	Positive peak intensity
Mothers	Left	0.73 *	0.80 *
	Right	0.74 *	0.19
Children	Left	0.60	0.57
	Right	0.28	0.14

* $P < 0.05$

Methods

Data acquisition

Magnetic fields were measured with the hyperscanning MEG system described above. Magnetic fields were sampled at 2000 Hz per channel (band pass filter 0.16-200 Hz for adults and children). The location of the head relative to the helmet of the MEG device was measured using 3 coils attached on the head surface as fiduciary points with respect to the landmarks (bilateral mastoid processes and nasion) for children, and using 5 coils for the mothers. After the MEG session, a three-dimensional digitizer (Polhemus FASTRAK, VT, USA) was used to digitize the head surface points and fiduciary landmarks of the mothers and children.

T1-weighted structural images (TR = 7 ms, TE = 3 ms, flip angle = 15°, field of view = 260 × 260 mm, matrix = 512 × 512 pixels, 166 slices with slice thickness = 1.2 mm) were acquired for all mothers using a Sigma Excite HD 1.5 T MRI system (GE Yokogawa). During the scan, five spherical lipid markers corresponding to the MEG fiduciary points were attached to the mother's head to allow for the superposition of the MEG coordinate system on the structural image.

Data Processing

Brain anatomical estimation for children

We could not obtain individual brain structural data because it is difficult for young children to perform MRI recordings without sedation. To superimpose the coordinate system of the MEG on the collected anatomical information, we estimated the brain structures from the individual head surface shapes in young children using the following methods, which are modified versions of our previous estimation algorithm [1]. Our algorithm was developed to find an optimal structural image from the 98 brain examples using the head surface points of a child.

The estimation of the brain structure consisted of the following three steps.

(1) We prepared a database of T1-weighted MR images from 98 children (age range: 0 – 8 years), which were regarded as templates of head surfaces and cortical structure for Japanese children. Then, for each of the above 98 template images, five fiduciary points (right preauricular, left preauricular, nasion, vertex, inion) on the head surface were determined.

(2) The root mean square error (RMSE) was calculated using the distance between the corresponding surface points of the child participants and a template. The RMSE was defined using the following formula:

$$\mathrm{RMSE} = \sqrt{\frac{1}{N}\sum_{i=1}^{N}\left(XC - XO\right)^2},$$

where XC are the coordinates of the child participant, XO are the coordinates of the template, and N is the number of surface points.

(3) The template with the lowest RMSEs of all templates was selected as an optimal brain template for the child participant.

Preprocessing for analysis of mu rhythm

First, blinking and cardiac data were identified using the Signal Space Projection (SSP) method for both the mothers and the children [2]. Second, any MEG data with motion noise were excluded based on the visual inspection of the waveforms and video recordings. Third, in the mothers, epochs with a magnetic amplitude greater than 3000 fT in all channels were automatically rejected to exclude data with motion noise or other noise contamination. In the children, epochs with a magnetic amplitude greater than 4000 fT were automatically rejected. Because of this preprocessing, we excluded the MEG data of 5 children due to excessive motion noise and excluded the MEG data of one mother due to excessive magnetic noise resulting from the presence of dental metals. In these excluded subjects, we could not obtain a sufficient period of noise-free MEG data (i.e., we captured less than a 50 sec period).

Source estimation

We performed signal source estimation of the mu rhythm using the individual anatomy of the mothers and using the individually estimated anatomy of their children. We focused on the upper mu band (10-12 Hz frequency band) in this study. Source reconstruction was performed with Brainstorm [3], which is documented and freely available for download online under the GNU general public license (http://neuroimage.usc.edu/brainstorm). To estimate the brain sources, we used an anatomically constrained MEG approach that places an anatomical constraint on the estimated sources by assuming that each individual's recorded brain activity lies in the cortical mantle [4]. In mothers, we used landmark information (right preauricular, left preauricular, nasion, vertex and inion) and digitized head surface points for MEG/MRI co-registration. The digitized head shape and the scalp surface of each individual were then used to reduce the minimum distance error between them in an iterative process. Cortical surfaces were created for each individual subject by automatically segmenting the T1-weighted MRIs into gray and white matter and defining the border between the

gray and white matter as the cortical surface. Cortical reconstruction and volumetric segmentation were performed in FreeSurfer (the open-source software: http://surfer.nmr.mgh.harvard.edu/) [5, 6]. Sensors were registered for each subject with the fiducial landmark using FASTRAK (Polhemus, VT, USA).

For MEG/MRI co-registration in the children, we employed the auditory evoked field (AEF) acquisition based on our previous study [7]. We matched the MEG and template MRI according to the location of AEF. Reconstruction of the MRI data and volumetric segmentation were performed in FreeSurfer.

The following procedure was common in mothers and children: the lead field was then computed using the overlapping spheres algorithm [8] with a cortical surface tessellated with 15000 vertices. The inverse solution was calculated for each individual using Tikhonov-regularized minimum-norm estimates [9]. A noise covariance matrix was calculated from MEG recordings from resting states (i.e., DVD condition) to estimate the noise level. Then, a weighted minimum-norm estimation with source orientation constraints was chosen to compute the source activity for mu rhythms. We used Desikan-Killiany atlases to estimate the region of interest [10].

Analysis of the behavioral contingency between mother and child

We analyzed the behavioral contingency between mother and child during face-to-face interactions using MEG data from the whole period of the “Live” condition, including motion noise. For correlation analysis with the index of mu suppression, the MEG data from eight mother-child pairs were analyzed. To quantify the mutual face-to-face interactions between mother and child, we calculated the correlation coefficient sequence between the magnitudes in the mother and child head motions (e.g., nod, nod back, nod no, etc.). The magnitudes of the head motions were quantified using motion artifacts in the MEG signals. Empirically, we regarded the MEG signals in the 0.2 - 0.5 Hz band as head motions during the face-to-face interactions. First, the 0.2 - 0.5 Hz band pass filter was applied to the whole time series MEG data of all channels. Second, the time series for the root-mean-squares (RMSs) of all channels were calculated. Third, the 0.1 - 0.3 Hz band pass filter was applied again to the RMS data to smooth sharp notches caused by the phase reversal of the magnetic field corresponding to the direction of the nod (e.g., the up and down direction). We addressed the processed time series data as head-motion-graphy (HMG) in the present study. As shown in supplementary movie S1, this HMG practically reflects head motions (e.g., nod, nod no, etc.). Fourth, the whole time series of the HMGs in mothers and children were segmented into 15 sec periods, which corresponded to the “Live” condition. The lengths

of the tolerable experimental periods differed among the pairs, and therefore, the mean number of 15 sec segment corresponding to the “Live” condition was actually 10.25 (range: 8 - 14). Fifth, the cross-correlation analysis between the time series of the HMGs in the mothers and children during the “Live” condition was completed for each 15 sec segment. Sixth, the time series of cross correlation coefficients calculated using these segments were averaged for each pair. Finally, we defined a peak value for the correlation coefficient within a 2 sec time window before and after time 0 as a strength of contingency, and we defined the latency of the peak as the direction of contingency (i.e., a positive value indicates that the mother is the leader, and a negative value indicates that the mother is the follower in terms of head movements during the face-to-face interactions). These offline analyses of the MEG data were performed with the Brain Vision analyzer (Brain Products GmbH, Gilching, Germany) and Matlab (MathWorks, Natick, MA).

References

1. Hayashi, N., *et al.* Algorithm for estimation of brain structural location from head surface shape in young children. *Neuroreport* **23**, 299-303 (2012).
2. Uusitalo, M.A. & Ilmoniemi, R.J. Signal-space projection method for separating MEG or EEG into components. *Medical & Biological Engineering & Computing* **35**, 135-140 (1997).
3. Tadel, F., Baillet, S., Mosher, J.C., Pantazis, D. & Leahy, R.M. Brainstorm: a user-friendly application for MEG/EEG analysis. *Comput Intell Neurosci* **2011**, 879716 (2011).
4. Dale, A.M., *et al.* Dynamic statistical parametric mapping: combining fMRI and MEG for high-resolution imaging of cortical activity. *Neuron* **26**, 55-67 (2000).
5. Dale, A.M., Fischl, B. & Sereno, M.I. Cortical surface-based analysis. I. Segmentation and surface reconstruction. *Neuroimage* **9**, 179-194 (1999).
6. Fischl, B., Sereno, M.I. & Dale, A.M. Cortical surface-based analysis. II: Inflation, flattening, and a surface-based coordinate system. *Neuroimage* **9**, 195-207 (1999).
7. Yoshimura, Y., *et al.* Language performance and auditory evoked fields in 2- to 5-year-old children. *Eur J Neurosci* **35**, 644-650 (2012).
8. Huang, M.X., Mosher, J.C. & Leahy, R.M. A sensor-weighted overlapping-sphere head model and exhaustive head model comparison for MEG. *Physics in medicine and biology* **44**, 423-440 (1999).
9. Baillet, S., Mosher, J.C. & Leahy, R.M. Electromagnetic brain mapping. *Ieee Signal Processing Magazine* **18**, 14-30 (2001).
10. Desikan, R.S., *et al.* An automated labeling system for subdividing the human cerebral cortex on MRI scans into gyral based regions of interest. *Neuroimage* **31**, 968-980 (2006).
